# High frequency of the D allele of the angiotensin-converting enzyme gene in Arabic populations

**DOI:** 10.1186/1756-0500-2-99

**Published:** 2009-06-08

**Authors:** Abdel Halim Salem, Mark A Batzer

**Affiliations:** 1 Department of Anatomy, College of Medicine and Medical Sciences, Arabian Gulf University, Manama, Kingdom of Bahrain; 2 Department of Anatomy, Faculty of Medicine, Suez Canal University, Ismailia, Egypt; 3 Department of Biological Sciences, 202 Life Sciences Building, Louisiana State University, Baton Rouge, LA 70803, USA

## Abstract

**Background:**

The angiotensin-converting enzyme (ACE) gene in humans has an insertion-deletion (I/D) polymorphic state in intron 16 on chromosome 17q23. This polymorphism has been widely investigated in different populations due to its association with the renin-angiotensin system. However, similar studies for Arab populations are limited. This study addresses the distribution of the ACE gene polymorphism in three Arab populations (Egyptians, Jordanians and Syrians).

**Findings:**

The polymorphisms of ACE gene were investigated using polymerase chain reaction for detection of an I/D mutation. The results showed a high frequency of the ACE *D *allele among the three Arab populations, Egyptians (0.67), Jordanians (0.66) and Syrians (0.60), which is similar to those obtained from previous studies for Arab populations.

**Conclusion:**

The relationship between ACE alleles and disease in these three Arab populations is still not known, but the present results clearly suggest that geographic origin should be carefully considered in the increasing number of studies on the association between ACE alleles and disease etiology. This study adds to the data showing the wide variation in the distribution of the ACE alleles in different populations and highlights that great care needs to be taken when interpreting clinical data on the association of the ACE alleles with different diseases.

## Background

Angiotensin-converting enzyme (ACE), a key enzyme of the rennin-angiotensin system, is localized in the kidney [1]. The ACE catalyzes the conversion of angiotensin I to the biologically active peptide, angiotensin II, which is involved in the control of fluid-electrolyte balance and systemic blood pressure [2]. The ACE gene is mapped to chromosome 17q23 and it has been widely investigated. The insertion/deletion (I/D) polymorphism of ACE was discovered by Rigat et al. [3] and it is characterized by the presence (insertion) or absence (deletion) of a 287 bp *Alu*Ya5 element inside intron 16 producing three genotypes (*II *homozygote, *ID *heterozygote and *DD *homozygote) [3]. Although the I/D polymorphism is located in a non-coding region (i.e. intron) of the ACE gene, several investigators have found that the *D *allele is related to increased activity of ACE in serum [3,4]. The highest serum ACE activity was seen in the *DD *genotype while the lowest was seen in the *II *genotype [3]. Several investigations suggested the genetic predisposition of the ACE I/D polymorphism with several diseases including coronary heart diseases [5], stroke [6], hypertension [7] and diabetes mellitus [8]. However, conflicting results have been reported regarding the association between ACE polymorphism and disease [9,10]. Moreover, various reports were published suggesting inter-ethnic variations in the frequency of allelic forms of the ACE genes [11,12].

In this study we aim to investigate the distribution of ACE gene I/D polymorphism in three Arab populations (Egyptians, Jordanians and Syrians). The three Arab populations have a mixed genetic background with an ethnic heterogeneity. Most of the three populations are of Mediterranean or Arabic origin that migrated from the Arabian Peninsula and surrounding areas.

## Methods

The human population samples used for this study have been described previously and were available from previous studies [13]. The samples studied were collected from unrelated individuals from three Arab populations: (Egyptians, Jordanians and Syrians) under institutionally approved internal review board protocols with informed consent. DNA was prepared from blood leukocytes by standard methods. A total of 164 Egyptians from Ismailia, and Sinai, 60 Jordanians and 70 Syrians were analyzed. The Egyptian samples were from Ismailia (112 subjects), and the Sinai (52 subjects). The specific segment of ACE gene was amplified by polymerase chain reaction (PCR) using the following primers [14]: ACE-F (5-CTGGAGACCACTCCCATCCTTTCT-3) and ACE-R (5-GATGTGGCCATCACATTCGTCAGAT-3). PCR amplification was carried out in 25 μl reactions containing 20–100 ng of template DNA, 40 pM of each oligonucleotide primers, 200 μM dNTPs, 50 mM KCl, 1.5 mM MgCl_2_, 10 mM Tris-HCl (pH 8.4) and *Taq *DNA polymerase (1.25 Units). The reaction were subjected to 32 cycles: an initial denaturation of 60 s at 94°C, 30 s denaturation at 94°C, 45 s at the annealing temperature 58°C, extension at 72°C for 45 s. Following the amplification cycles, a final extension was performed at 72°C for 10 min. For analysis, 20 μl of each sample was fractionated on a 2% agarose gel with 0.05 μg/ml ethidium bromide. PCR products were directly visualized using UV fluorescence. The homozygous individuals for the *D *allele (*DD *genotype) were identified by the presence of a single 190 bp PCR product. The homozygous for the *I *allele (*II *genotype) were identified by the presence of a single 490 bp PCR product. The heterozygous individuals (*ID *genotype) were identified by the presence of both 190 and 490 bp PCR products. Because the *D *allele in heterozygous samples is preferentially amplified, all samples that were typed initially as a *DD *genotype were reanalyzed using an insertion-specific primer pair, as reported by Lindpaintner et al. [15], except that the annealing temperature was 67°C. A 335 bp band was obtained only in the presence of the *I *allele and no bands were detected for samples with *DD *genotype.

Statistical analysis was performed using SPSS version 15 statistical package for windows. Allele and genotype frequencies were calculated by direct counting; the Hardy-Weinberg equilibrium was assessed by an exact test provided by the Arlequin program [16].

## Results and discussion

As shown in Table 1, 16 individuals from Egypt living in Ismailia were homozygous for the *II *genotype, 40 were heterozygous for the *ID *genotype and 56 were homozygous for the *DD *genotype, giving a *D *allelic frequency of 0.679. Among 52 Egyptians living in Sinai that were studied, 4 individuals were homozygous for the *II *genotype, 27 were heterozygous for the *ID *genotype and 21 were homozygous for the *DD *genotype, giving a *D *allelic frequency of 0.663. There was no significant statistical difference between the two Egyptian groups (X^2 ^= 4.25, P-value = 0.119). The average frequency of the *D *allele among the two studied Egyptian groups was 0.674. As regards the studied individuals from Syria, we found that 9 individuals were homozygous for the *II *genotype, 38 were heterozygous for the *ID *genotype and 23 were homozygous for the *DD *genotype, giving a *D *allelic frequency of 0.600. As regards the Jordanians, we found that 13 individuals were homozygous for the *II *genotype, 15 were heterozygous for the *ID *genotype and 32 were homozygous for the *DD *genotype, giving a *D *allelic frequency of 0.658. Based on the allele frequencies, it is possible to predict the genotype frequency considering that they follow the Hardy-Weinberg equilibrium. This means that the frequencies have a binomial distribution according to the following equation: *p*^2^+2*p*q + *q*^2 ^= 1, where p and q are the allelic frequencies of *I *and *D*, respectively, and *p*^2^, 2*p*q and *q*^2 ^are the genotype frequencies of *II*, *ID*, and *DD*, respectively. No significant deviations from the Hardy-Weinberg equilibrium were observed except for Jordanians which probably represent a random statistical fluctuation. Table 2 shows the frequency of the *D *allele in the populations analyzed here as well as selected populations from previous studies.

**Table 1:** Allele frequencies and heterozygosities of the ACE gene among Egyptians, Jordanians and Syrians.

				**ACE Allele Frequency**		**Heterozygosity**	
				
**Population**	**N**	**ACE Genotype**	**Number Observed** **(and Expected)**	***I***	***D***	**Observed**	**Expected**
Egypt-Ismailia	112	*II*	16 (13.66)	0.321	0.679	0.357	0.474
		*ID*	40 (45.76)				
		*DD*	56 (52.59)				
Egypt-Sinai	52	*II*	4 (6.34)	0.337	0.663	0.519	0.474
		*ID*	27 (21.24)				
		*DD*	21 (24.42)				
Egypt-Total	164	*II*	20 (17.43)	0.326	0.674	0.409	0.473
		*ID*	67 (72.07)				
		*DD*	77 (74.50)				
Syrians	70	*II*	9 (11.20)	0.400	0.600	0.543	0.527
		*ID*	38 (33.60)				
		*DD*	23 (25.20)				
Jordanians	60	*II*	13 (7.01)	0.342	0.658	0.250	0.505
		*ID*	15 (25.9)				
		*DD*	32 (27)				

**Table 2:** The frequency of I/D polymorphism of the ACE gene in the current study compared to different ethnic groups in different studies.

	**Allele Frequency**		
		
**Ethnic Group**	***I***	***D***	**Number of Individuals**
Tunisians [17]	0.24	0.76	47
Algerians [17]	0.27	0.73	48
Somalis [18]	0.27	0.73	53
Omanis [18]	0.29	0.71	127
Egyptians [20]	0.28	0.72	188
Moroccans [17]	0.30	0.70	300
Egyptians ^[present study]^	0.33	0.67	164
Jordanians ^[present study]^	0.34	0.66	60
Emiratis [19]	0.34	0.66	159
Sudanese [18]	0.36	0.64	121
Emiratis [18]	0.39	0.61	111
Syrians ^[present study]^	0.40	0.60	70
Nigerians [11]	0.41	0.59	80
Caucasians [11]	0.49	0.51	186
Caucasians [21]	0.54	0.46	733
Indians [12]	0.54	0.46	166
Japanese [22]	0.65	0.35	136
Japanese [23]	0.67	0.33	113
Chinese [24]	0.71	0.29	189
Yanomami Indians [11]	0.85	0.15	49
Samoans [11]	0.91	0.09	58
Australian Aborigines [25]	0.97	0.03	184

Data from previous studies is denoted by the citations.

The present study investigated for the first time, the frequency of the ACE gene I/D polymorphism in randomly selected Syrian and Jordanian individuals. The frequency of the *D *allele of the ACE gene (Table 2) among Syrians (0.60), Jordanians (0.66) and Egyptians from Ismailia (0.68), and from Sinai (0.66) is similar to that in other Arabs, such as the Tunisians (0.76) [17], the Algerians (0.73) [17], the Somalis (0.73) [18], the Omanis (0.71) [18], the Moroccans (0.70) [17], the Emiratis (0.61–0.66) [18,19], and the Sudanese (0.64) [18]. Our result for the Egyptian samples was slightly different from previously reported *D *allele frequency for an Egyptian population sample from Cairo [20]. This difference most likely is due to the composition of the samples used in the two studies. Overall similarity in the I/D allele's frequencies of the Egyptian, Jordanian and Syrian population samples studied with other Arab populations suggests that there may have been some admixture among them.

Compared to other geographic groups (Table 2) the frequency of the *D *allele in the Arab populations is among the highest reported. The frequency of the *D *allele is highest among sub-Saharan Africans [11] and Arabs (0.60–0.76) [17-20], moderate for Caucasians (0.46–0.51) [11,21], and low among various Asian populations (0.29–0.46) [12,22-24]. The Yanomami Indians, Samoans and Australian Aborigines seem to have the lowest frequencies: 0.15, 0.09 and 0.03, respectively [11,25].

The worldwide distribution of the *D *allele (Table 2) suggests that the ancestral state present in the human population was the *D *allele and that an *Alu*Ya5 (the youngest *Alu*Y subfamily in the human genome) element later inserted at the locus, generating the *I *allele and creating an ancestral polymorphism in the pre-migration African human population. As human populations moved out of Africa during Paleolithic migrations 100,000 years ago [26], they carried with them portions of this ancestral I/D polymorphism. As each different human population migrated to its new location and established itself, they were subject to different evolutionary forces (e.g., genetic drift, selective pressure, founder effects, and gene flow), which shaped the allele frequencies we observe in these populations today.

Although the I/D polymorphism in the ACE gene implies either an insertion or a deletion, there is only an insertion event, and the *D *allele represents the ancestral state of the ACE gene without an *Alu *repeat insertion in the region of intron 16 [14]. In fact, nearly half of the human genome is derived from transposable elements, and among them, the primate-specific *Alu *elements are most abundant, accounting for more than 10% of the human genome.

In conclusion, the results of the distribution of the ACE I/D gene polymorphism obtained for the *D *allele among Egyptians, Jordanians and Syrians are comparable to those obtained from previous studies in other Arabs, add to the data indicating the wide variations observed in the frequency of the ACE alleles among the peoples of the world and highlights that great care needs to be taken when interpreting clinical data on the association of the ACE alleles with different diseases.

## Competing interests

The authors declare that they have no competing interests.

## Authors' contributions

ASH designed the research project. ASH performed the experiments and statistical analysis. MAB contributed reagents/materials/analytic tools. AHS and MAB wrote the manuscript. All authors have read and approved the final manuscript.
